# Residual lung abnormality following COVID-19 hospitalisation is characterised by biomarkers of epithelial injury

**DOI:** 10.1016/j.ebiom.2026.106134

**Published:** 2026-01-24

**Authors:** Iain Stewart, Joseph Jacob, Joanna C. Porter, Bin Liu, Amanda L. Tatler, Nancy Gomez, Matthew R. Pugh, Alison E. John, Richard J. Allen, John F. Blaikley, Nazia Chaudhuri, Emma Denneny, Laura Fabbri, Peter M. George, Beatriz Guillen-Guio, Bibek Gooptu, Ian P. Hall, Ling Pei Ho, Ian Jarrold, Simon Johnson, Mark G. Jones, Fasihul Khan, Puja Mehta, Jane Mitchell, Philip L. Molyneaux, John E. Pearl, Karen Piper Hanley, Manuela Platé, Valerie Quinn, Pilar Rivera-Ortega, Laura C. Saunders, David J.F. Smith, Mark Spears, Lisa G. Spencer, Stefan C. Stanel, A.A. Roger Thompson, Simon Walsh, Jim M. Wild, Dan G. Wootton, Annemarie B. Docherty, Fergus Gleeson, William Greenhalf, Ewen M. Harrison, Nazir Lone, Jennifer Quint, Anastasia Maslova, Moritz Pohl, Adam Stephens, Simon Young, Amisha Singapuri, Aarti Shikotra, Marco Sereno, Ruth M. Saunders, Matthew Richardson, Betty Raman, Krisnah Poinasamy, Hamish J.C. McAuley, Michael Marks, Olivia C. Leavy, Linzy Houchen-Wolloff, Alex Horsley, Victoria C. Harris, Neil Greening, Rachael A. Evans, Omer Elneima, James D. Chalmers, Christopher E. Brightling, Rachel C. Chambers, Louise V. Wain, R. Gisli Jenkins, PHOSP-COVID Collaborative Group and the UKILD Consortium, K. Abel, H. Adamali, D. Adeloye, O. Adeyemi, R. Adrego, L.A. Aguilar Jimenez, S. Ahmad, N. Ahmad Haider, R. Ahmed, N. Ahwireng, M. Ainsworth, B. Al-Sheklly, A. Alamoudi, M. Ali, M. Aljaroof, A.M. All, L. Allan, R.J. Allen, L. Allerton, L. Allsop, P. Almeida, D. Altmann, M. Alvarez Corral, S. Amoils, D. Anderson, C. Antoniades, G. Arbane, A. Arias, C. Armour, L. Armstrong, N. Armstrong, D. Arnold, H. Arnold, A. Ashish, A. Ashworth, M. Ashworth, S. Aslani, H. Assefa-Kebede, C. Atkin, P. Atkin, R. Aul, H. Aung, L. Austin, C. Avram, A. Ayoub, M. Babores, R. Baggott, J. Bagshaw, D. Baguley, L. Bailey, J.K. Baillie, S. Bain, M. Bakali, M. Bakau, E. Baldry, D. Baldwin, M. Baldwin, C. Ballard, A. Banerjee, B. Bang, R.E. Barker, L. Barman, S. Barratt, F. Barrett, D. Basire, N. Basu, M. Bates, A. Bates, R. Batterham, H. Baxendale, H. Bayes, M. Beadsworth, P. Beckett, M. Beggs, M. Begum, P. Beirne, D. Bell, R. Bell, K. Bennett, E. Beranova, A. Bermperi, A. Berridge, C. Berry, S. Betts, E. Bevan, K. Bhui, M. Bingham, K. Birchall, L. Bishop, K. Bisnauthsing, J. Blaikely, A. Bloss, A. Bolger, C.E. Bolton, J. Bonnington, A. Botkai, C. Bourne, M. Bourne, K. Bramham, L. Brear, G. Breen, J. Breeze, A. Briggs, E. Bright, C.E. Brightling, S. Brill, K. Brindle, L. Broad, A. Broadley, C. Brookes, M. Broome, A. Brown, A. Brown, J. Brown, J. Brown, J.S. Brown, M. Brown, M. Brown, V. Brown, T. Brugha, N. Brunskill, M. Buch, P. Buckley, A. Bularga, E. Bullmore, L. Burden, T. Burdett, D. Burn, G. Burns, A. Burns, J. Busby, R. Butcher, A. Butt, S. Byrne, P. Cairns, P.C. Calder, E. Calvelo, H. Carborn, B. Card, C. Carr, L. Carr, G. Carson, P. Carter, A. Casey, M. Cassar, J. Cavanagh, M. Chablani, T. Chalder, J.D. Chalmers, R.C. Chambers, F. Chan, K.M. Channon, K. Chapman, A. Charalambou, N. Chaudhuri, A. Checkley, J. Chen, Y. Cheng, L. Chetham, C. Childs, E.R. Chilvers, H. Chinoy, A. Chiribiri, K. Chong-James, G. Choudhury, N. Choudhury, P. Chowienczyk, C. Christie, M. Chrystal, D. Clark, C. Clark, J. Clarke, S. Clohisey, G. Coakley, Z. Coburn, S. Coetzee, J. Cole, C. Coleman, F. Conneh, D. Connell, B. Connolly, L. Connor, A. Cook, B. Cooper, J. Cooper, S. Cooper, D. Copeland, T. Cosier, M. Coulding, C. Coupland, E. Cox, T. Craig, P. Crisp, D. Cristiano, M.G. Crooks, A. Cross, I. Cruz, P. Cullinan, D. Cuthbertson, L. Daines, M. Dalton, P. Daly, A. Daniels, P. Dark, J. Dasgin, A. David, C. David, E. Davies, F. Davies, G. Davies, G.A. Davies, K. Davies, M.J. Davies, J. Dawson, E. Daynes, A. De Soyza, B. Deakin, A. Deans, C. Deas, J. Deery, S. Defres, A. Dell, K. Dempsey, E. Denneny, J. Dennis, A. Dewar, R. Dharmagunawardena, N. Diar-Bakerly, C. Dickens, A. Dipper, S. Diver, S.N. Diwanji, M. Dixon, R. Djukanovic, H. Dobson, S.L. Dobson, A.B. Docherty, A. Donaldson, T. Dong, N. Dormand, A. Dougherty, R. Dowling, S. Drain, K. Draxlbauer, K. Drury, H.J.C. drury, P. Dulawan, A. Dunleavy, S. Dunn, C. Dupont, J. Earley, N. Easom, C. Echevarria, S. Edwards, C. Edwardson, H. El-Taweel, A. Elliott, K. Elliott, Y. Ellis, A. Elmer, O. Elneima, D. Evans, H. Evans, J. Evans, R. Evans, R.A. Evans, R.I. Evans, T. Evans, C. Evenden, L. Evison, L. Fabbri, S. Fairbairn, A. Fairman, K. Fallon, D. Faluyi, C. Favager, T. Fayzan, J. Featherstone, T. Felton, J. Finch, S. Finney, J. Finnigan, L. Finnigan, H. Fisher, S. Fletcher, R. Flockton, M. Flynn, H. Foot, D. Foote, A. Ford, D. Forton, E. Fraile, C. Francis, R. Francis, S. Francis, A. Frankel, E. Fraser, R. Free, N. French, X. Fu, J. Fuld, J. Furniss, L. Garner, N. Gautam, J.R. Geddes, J. George, P. George, M. Gibbons, M. Gill, L. Gilmour, F. Gleeson, J. Glossop, S. Glover, N. Goodman, C. Goodwin, B. Gooptu, H. Gordon, T. Gorsuch, M. Greatorex, P.L. Greenhaff, W. Greenhalf, A. Greenhalgh, N.J. Greening, J. Greenwood, H. Gregory, R. Gregory, D. Grieve, D. Griffin, L. Griffiths, A.-M. Guerdette, B. Guillen Guio, M. Gummadi, A. Gupta, S. Gurram, E. Guthrie, Z. Guy, H.H. Henson, K. Hadley, A. Haggar, K. Hainey, B. Hairsine, P. Haldar, I. Hall, L. Hall, M. Halling-Brown, R. Hamil, A. Hancock, K. Hancock, N.A. Hanley, S. Haq, H.E. Hardwick, E. Hardy, T. Hardy, B. Hargadon, K. Harrington, E. Harris, V.C. Harris, E.M. Harrison, P. Harrison, N. Hart, A. Harvey, M. Harvey, M. Harvie, L. Haslam, M. Havinden-Williams, J. Hawkes, N. Hawkings, J. Haworth, A. Hayday, M. Haynes, J. Hazeldine, T. Hazelton, L.G. Heaney, C. Heeley, J.L. Heeney, M. Heightman, S. Heller, M. Henderson, L. Hesselden, M. Hewitt, V. Highett, T. Hillman, T. Hiwot, L.P. Ho, A. Hoare, M. Hoare, J. Hockridge, P. Hogarth, A. Holbourn, S. Holden, L. Holdsworth, D. Holgate, M. Holland, L. Holloway, K. Holmes, M. Holmes, B. Holroyd-Hind, L. Holt, A. Hormis, A. Horsley, A. Hosseini, M. Hotopf, L. Houchen-Wolloff, K. Howard, L.S. Howard, A. Howell, E. Hufton, A.D. Hughes, J. Hughes, R. Hughes, A. Humphries, N. Huneke, E. Hurditch, J. Hurst, M. Husain, T. Hussell, J. Hutchinson, W. Ibrahim, F. Ilyas, J. Ingham, L. Ingram, D. Ionita, K. Isaacs, K. Ismail, T. Jackson, J. Jacob, W.Y. James, W. Jang, C. Jarman, I. Jarrold, H. Jarvis, R. Jastrub, B. Jayaraman, R.G. Jenkins, P. Jezzard, K. Jiwa, C. Johnson, S. Johnson, D. Johnston, C.J. Jolley, D. Jones, G. Jones, H. Jones, H. Jones, I. Jones, L. Jones, M.G. Jones, S. Jones, S. Jose, T. Kabir, G. Kaltsakas, V. Kamwa, N. Kanellakis, S. Kaprowska, Z. Kausar, N. Keenan, S. Kelly, G. Kemp, S. Kerr, H. Kerslake, A.L. Key, F. Khan, K. Khunti, S. Kilroy, B. King, C. King, L. Kingham, J. Kirk, P. Kitterick, P. Klenerman, L. Knibbs, S. Knight, A. Knighton, O. Kon, S. Kon, S.S. Kon, S. Koprowska, A. Korszun, I. Koychev, C. Kurasz, P. Kurupati, C. Laing, H. Lamlum, G. Landers, C. Langenberg, D. Lasserson, L. Lavelle-Langham, A. Lawrie, C. Lawson, C. Lawson, A. Layton, A. Lea, O.C. Leavy, D. Lee, J.-H. Lee, E. Lee, K. Leitch, R. Lenagh, D. Lewis, J. Lewis, K.E. Lewis, V. Lewis, N. Lewis–Burke, X. Li, T. Light, L. Lightstone, W. Lilaonitkul, L. Lim, S. Linford, A. Lingford-Hughes, M. Lipman, K. Liyanage, A. Lloyd, S. Logan, D. Lomas, N.I. Lone, R. Loosley, J.M. Lord, H. Lota, W. Lovegrove, A. Lucey, E. Lukaschuk, A. Lye, C. Lynch, S. MacDonald, G. MacGowan, I. Macharia, J. Mackie, L. Macliver, S. Madathil, G. Madzamba, N. Magee, M.M. Magtoto, N. Mairs, N. Majeed, E. Major, F. Malein, M. Malim, G. Mallison, W.D.-C. Man, S. Mandal, K. Mangion, C. Manisty, R. Manley, K. March, S. Marciniak, P. Marino, M. Mariveles, M. Marks, E. Marouzet, S. Marsh, B. Marshall, M. Marshall, J. Martin, A. Martineau, L.M. Martinez, N. Maskell, D. Matila, W. Matimba-Mupaya, L. Matthews, A. Mbuyisa, S. McAdoo, H. McAllister-Williams, A. McArdle, P. McArdle, D. McAulay, G.P. McCann, J. McCormick, W. McCormick, P. McCourt, L. McGarvey, C. McGee, K. Mcgee, J. McGinness, K. McGlynn, A. McGovern, H. McGuinness, I.B. McInnes, J. McIntosh, E. McIvor, K. McIvor, L. McLeavey, A. McMahon, M.J. McMahon, L. McMorrow, T. Mcnally, M. McNarry, J. McNeill, A. McQueen, H. McShane, C. Mears, C. Megson, S. Megson, P. Mehta, J. Meiring, L. Melling, M. Mencias, D. Menzies, M. Merida Morillas, A. Michael, C. Miller, L. Milligan, C. Mills, G. Mills, N.L. Mills, L. Milner, S. Misra, J. Mitchell, A. Mohamed, N. Mohamed, S. Mohammed, P.L. Molyneaux, W. Monteiro, S. Moriera, A. Morley, L. Morrison, R. Morriss, A. Morrow, A.J. Moss, P. Moss, K. Motohashi, N. Msimanga, E. Mukaetova-Ladinska, U. Munawar, J. Murira, U. Nanda, H. Nassa, M. Nasseri, A. Neal, R. Needham, P. Neill, S. Neubauer, D.E. Newby, H. Newell, T. Newman, J. Newman, A. Newton–Cox, T. Nicholson, D. Nicoll, A. Nikolaidis, C.M. Nolan, M.J. Noonan, C. Norman, P. Novotny, J. Nunag, L. Nwafor, U. Nwanguma, J. Nyaboko, C. O'Brien, K. O'Donnell, D. O'Regan, L. O'Brien, N. Odell, G. Ogg, O. Olaosebikan, C. Oliver, Z. Omar, P.J.M. Openshaw, L. Orriss-Dib, L. Osborne, R. Osbourne, M. Ostermann, C. Overton, J. Owen, J. Oxton, J. Pack, E. Pacpaco, S. Paddick, S. Painter, A. Pakzad, S. Palmer, P. Papineni, K. Paques, K. Paradowski, M. Pareek, D. Parekh, H. Parfrey, C. Pariante, S. Parker, M. Parkes, J. Parmar, S. Patale, B. Patel, M. Patel, S. Patel, D. Pattenadk, M. Pavlides, S. Payne, L. Pearce, J.E. Pearl, D. Peckham, J. Pendlebury, Y. Peng, C. Pennington, I. Peralta, E. Perkins, Z. Peterkin, T. Peto, N. Petousi, J. Petrie, P. Pfeffer, J. Phipps, J. Pimm, K. Piper Hanley, R. Pius, H. Plant, S. Plein, T. Plekhanova, M. Plowright, K. Poinasamy, O. Polgar, L. Poll, J.C. Porter, J. Porter, S. Portukhay, N. Powell, A. Prabhu, J. Pratt, A. Price, C. Price, C. Price, D. Price, L. Price, L. Price, A. Prickett, J. Propescu, S. Prosper, S. Pugmire, S. Quaid, J. Quigley, J. Quint, H. Qureshi, I.N. Qureshi, K. Radhakrishnan, N.M. Rahman, M. Ralser, B. Raman, A. Ramos, H. Ramos, J. Rangeley, B. Rangelov, L. Ratcliffe, P. Ravencroft, A. Reddington, R. Reddy, A. Reddy, H. Redfearn, D. Redwood, A. Reed, M. Rees, T. Rees, K. Regan, W. Reynolds, C. Ribeiro, A. Richards, E. Richardson, M. Richardson, P. Rivera-Ortega, K. Roberts, E. Robertson, E. Robinson, L. Robinson, L. Roche, C. Roddis, J. Rodger, A. Ross, G. Ross, J. Rossdale, A. Rostron, A. Rowe, A. Rowland, J. Rowland, M.J. Rowland, S.L. Rowland–Jones, K. Roy, M. Roy, I. Rudan, R. Russell, E. Russell, G. Saalmink, R. Sabit, E.K. Sage, T. Samakomva, N. Samani, C. Sampson, K. Samuel, R. Samuel, A. Sanderson, E. Sapey, D. Saralaya, J. Sargant, C. Sarginson, T. Sass, N. Sattar, K. Saunders, R.M. Saunders, P. Saunders, L.C. Saunders, H. Savill, W. Saxon, A. Sayer, J. Schronce, W. Schwaeble, J.T. Scott, K. Scott, N. Selby, M.G. Semple, M. Sereno, T.A. Sewell, A. Shah, K. Shah, P. Shah, M. Shankar-Hari, M. Sharma, C. Sharpe, M. Sharpe, S. Shashaa, A. Shaw, K. Shaw, V. Shaw, A. Sheikh, S. Shelton, L. Shenton, K. Shevket, A. Shikotra, J. Short, S. Siddique, S. Siddiqui, J. Sidebottom, L. Sigfrid, G. Simons, J. Simpson, N. Simpson, A. Singapuri, C. Singh, S. Singh, S.J. Singh, D. Sissons, J. Skeemer, K. Slack, A. Smith, D. Smith, S. Smith, J. Smith, L. Smith, M. Soares, T.S. Solano, R. Solly, A.R. Solstice, T. Soulsby, D. Southern, D. Sowter, M. Spears, L.G. Spencer, F. Speranza, L. Stadon, S. Stanel, N. Steele, M. Steiner, D. Stensel, G. Stephens, L. Stephenson, M. Stern, I. Stewart, R. Stimpson, S. Stockdale, J. Stockley, W. Stoker, R. Stone, W. Storrar, A. Storrie, K. Storton, E. Stringer, S. Strong-Sheldrake, N. Stroud, C. Subbe, C.L. Sudlow, Z. Suleiman, C. Summers, C. Summersgill, D. Sutherland, D.L. Sykes, R. Sykes, N. Talbot, A.L. Tan, L. Tarusan, V. Tavoukjian, A. Taylor, C. Taylor, J. Taylor, A. Te, H. Tedd, C.J. Tee, J. Teixeira, H. Tench, S. Terry, S. Thackray-Nocera, F. Thaivalappil, B. Thamu, D. Thickett, C. Thomas, D.C. Thomas, S. Thomas, A.K. Thomas, T. Thomas-Woods, T. Thompson, A.A.R. Thompson, T. Thornton, M. Thorpe, R.S. Thwaites, J. Tilley, N. Tinker, G.F. Tiongson, M. Tobin, J. Tomlinson, C. Tong, M. Toshner, R. Touyz, K.A. Tripp, E. Tunnicliffe, A. Turnbull, E. Turner, S. Turner, V. Turner, K. Turner, S. Turney, L. Turtle, H. Turton, J. Ugoji, R. Ugwuoke, R. Upthegrove, J. Valabhji, M. Ventura, J. Vere, C. Vickers, B. Vinson, E. Wade, P. Wade, L.V. Wain, T. Wainwright, L.O. Wajero, S. Walder, S. Walker, S. Walker, E. Wall, T. Wallis, S. Walmsley, J.A. Walsh, S. Walsh, L. Warburton, T.J.C. Ward, K. Warwick, H. Wassall, S. Waterson, E. Watson, L. Watson, J. Watson, J. Weir McCall, C. Welch, H. Welch, B. Welsh, S. Wessely, S. West, H. Weston, H. Wheeler, S. White, V. Whitehead, J. Whitney, S. Whittaker, B. Whittam, V. Whitworth, A. Wight, J. Wild, M. Wilkins, D. Wilkinson, B. Williams, N. Williams, N. Williams, J. Williams, S.A. Williams–Howard, M. Willicombe, G. Willis, J. Willoughby, A. Wilson, D. Wilson, I. Wilson, N. Window, M. Witham, R. Wolf-Roberts, C. Wood, F. Woodhead, J. Woods, D.G. Wootton, J. Wormleighton, J. Worsley, D. Wraith, C. Wrey Brown, C. Wright, L. Wright, S. Wright, J. Wyles, I. Wynter, M. Xu, N. Yasmin, S. Yasmin, T. Yates, K.P. Yip, B. Young, S. Young, A. Young, A.J. Yousuf, A. Zawia, L. Zeidan, B. Zhao, B. Zheng, O. Zongo

**Affiliations:** a National Heart and Lung Institute, Imperial College London, UK; b NIHR Imperial Biomedical Research Centre, Imperial College London, UK; c Satsuma Lab, Centre for Medical Imaging Computing, University College London, UK; d UCL Respiratory, University College London, UK; e University College London Hospitals NHS Foundation Trust, UK; f Department of Health Sciences, University of Leicester, UK; g NIHR Leicester Biomedical Research Centre, University of Leicester, UK; h Manchester University NHS Foundation Trust, UK; i School of Biological Sciences, University of Manchester, UK; j Ulster University, UK; k Royal Brompton and Harefield Clinical Group, Guy's and St Thomas' NHS Foundation Trust, UK; l School of Medicine, University of Nottingham, UK; m NIHR Nottingham Biomedical Research Centre, University of Nottingham, UK; n Oxford University Hospitals NHS Foundation Trust, UK; o Respiratory Medicine Unit, Nuffield Department of Medicine, University of Oxford, UK; p Asthma + Lung UK, UK; q University Hospital Southampton NHS Foundation Trust, UK; r University Hospitals of Leicester NHS Trust, UK; s Department of Respiratory Sciences, University of Leicester, UK; t School of Medical Sciences, University of Manchester, UK; u School of Medicine & Population Health, University of Sheffield, UK; v NHS Tayside & University of Dundee, UK; w Liverpool University Hospitals NHS Foundation Trust, UK; x NIHR Sheffield Biomedical Research Centre, University of Sheffield, UK; y Centre for Medical Informatics, The Usher Institute, University of Edinburgh, UK; z University of Liverpool, UK; aa Sysmex R&D Centre UK, UK; ab London School of Hygiene & Tropical Medicine, UK; ac Institute of Immunology and Immunotherapy, University of Birmingham, UK

**Keywords:** COVID-19, Hospitalisation, Lung injury, Biomarker, Epithelial

## Abstract

**Background:**

Long term respiratory symptoms are reported following recovery of acute COVID-19 infection and residual lung abnormalities (RLA) on follow-up thoracic computed tomography (CT) after COVID-19 hospitalisation have been observed. It is unknown whether RLA are associated with epithelial lung injury.

**Methods:**

Plasma was sampled from the observational Post HOSPitalisation-COVID cohort at five months post-hospitalisation. Epithelial injury biomarkers Krebs von den Lungen-6 (KL-6), matrix metalloproteinase 7 (MMP-7), surfactant protein-D (SP-D) and surfactant protein-A (SP-A) were assayed. In those without follow-up CT, RLA at-risk was defined by percent predicted DL_CO_ <80% and/or abnormal chest X-ray, otherwise they were considered low-risk. Follow-up CT RLA was defined as combined involvement of ground glass opacity and reticulation ≥10%.

**Findings:**

A total of 957 people were included, 846 people with no CT (at-risk n = 103; 12.2%), 111 people with follow-up CT (RLA ≥10% n = 85; 76.6%). All epithelial injury biomarkers were significantly elevated in people at-risk of RLA compared with low-risk. KL-6 and MMP-7 were significantly higher in people with ≥10% RLA than those with <10%, SP-D and SP-A did not reach significance. SP-D and SP-A were associated with percent involvement of reticulation (3.22%, 95% CI 1.19–5.24; 3.03%, 95% CI 0.76–5.30, respectively).

**Interpretation:**

RLA after acute COVID-19 infection were consistent with elevated epithelial injury biomarkers and pro-fibrotic signalling. Future studies should address the temporal association between fibrotic biomarkers and resolution or progression of radiological involvement.

**Funding:**

MRC-UK Research and Innovation and 10.13039/501100000272National Institute for Health Research (NIHR) rapid response panel to tackle COVID-19 (MR/V027859/1; COV0319; MR/W006111/1).

**Research in context**

**Evidence before this study**

Some survivors of acute COVID-19 infection have long-term symptoms that could suggest ongoing lung impairment. Searches performed in MEDLINE and Embase for SARS-COV-2 studies with radiological lung follow-up estimated that 50% of participants had inflammatory patterns and 29% had fibrotic patterns at a median of 3 months post infection. Analysis of the UK nationwide Post-hospitalisation COVID-19 Study at 5-months follow-up suggested that up to 11% of people discharged from hospital following COVID-19 infection were at-risk of radiological residual lung abnormalities, such as ground glass opacity and reticulation. In people with pulmonary fibrosis, these radiological patterns are often consistent with persistent epithelial lung injury. Biomarker studies have identified associations with COVID-19 severity, however there are few studies that explore the relationship between biomarkers of epithelial injury and parenchymal lung abnormalities post-hospitalisation.

**Added value of this study**

Using the Post-hospitalisation COVID-19 Study at five months follow-up, we present analysis of four biomarkers of epithelial lung injury that have been validated in pulmonary fibrosis. We compare levels according to the risk of residual lung abnormality in 846 people defined according to dysfunctional gas exchange and abnormal chest X-ray. Greater levels of matrix metalloproteinase-7 and Krebs von den Lungen-6 were observed in participants at-risk of residual lung abnormality, which was consistent in an internal replication cohort of 111 people with evidence from thoracic CT. We report epithelial injury biomarker associations according to the extent of radiological abnormalities and demonstrate that surfactant proteins were associated with reticulation and not ground glass opacity. Associations of epithelial injury biomarkers with radiological abnormalities were independent of age, sex and admission severity. We observed greater gene and protein expression levels of epithelial injury biomarkers in epithelial cells from COVID-19 lung tissue.

**Implications of all the available evidence**

These findings provide evidence that raised, circulating levels of epithelial lung injury biomarkers were observed five months following hospitalisation due to COVID-19, were associated with the extent of radiological abnormality, and may be useful for monitoring people at-risk of parenchymal lung changes. The results suggests that pro-fibrotic signalling cascades contribute to persistent epithelial lung injury observed in some people recovering from COVID-19, which may contribute to ongoing symptoms and restrictive lung function. Further research and surveillance are required to address the longitudinal association of epithelial lung injury biomarkers and radiological patterns following COVID-19 in order to understand whether fibrotic features are stable, resolving or progressive in the long-term.

## Introduction

Following acute COVID-19 infection, long-term symptoms have been frequently reported by survivors.^1^ Meta-analysis of post-COVID-19 lung sequelae has highlighted dyspnoea at a prevalence estimate of 37%,^2^ impaired gas transfer at 38%, restrictive impairment at 17%, thoracic computed tomography (CT) evidence of fibrotic involvement at 29% and inflammatory involvement at 50%.^3^ These sequelae are consistent with lung injury that for some is chronic and can be associated with a poor recovery following acute COVID-19 infection.

The Post-Hospitalisation COVID-19 (PHOSP-COVID) study was designed to identify associations with good or poor recovery,^4^ with less than 30% of participants reporting feeling fully recovered at one year.^5^ Risk factors for poor recovery of breathlessness included older age, obesity, major comorbidities, and length of acute admission.^6^ The UK interstitial lung disease (ILD) Post-COVID study previously leveraged the PHOSP-COVID dataset to assess the prevalence and risk factors of residual lung abnormalities (RLA) on thoracic CT in people without evidence of prior clinical management for ILD.^7^^,^^8^ Important clinical risk factors for RLA included the need for ventilation during hospitalisation, abnormal follow-up chest X-ray, and impaired gas transfer at five months post-hospitalisation, with up to 11% of survivors estimated to be at-risk of RLA.

Mechanisms of parenchymal lung changes include persistent epithelial lung injury that can result in aberrant wound healing and deposition of extracellular matrix components, with validated biomarkers that are elevated in fibrotic lung disease including matrix metalloproteinase-7 (MMP-7), Krebs von den Lungen-6 (KL-6), surfactant protein-A (SP-A), and surfactant protein-D (SP-D).^9^, ^10^, ^11^, ^12^, ^13^, ^14^, ^15^ Whilst the UK-ILD Post-COVID evidence of RLA is consistent with other studies,^16^^,^^17^ it is unknown whether RLA represents sequelae that will resolve over time or lead to persistent epithelial lung injury that may be consistent with interstitial lung changes.^18^

The aim of this UK ILD Post-COVID study was to assess whether biomarker levels of epithelial lung injury were greater in people at-risk of RLA and whether this effect was consistent in participants with evidence of RLA involvement on CT.

## Methods

### Study population

The study cohort included participants of the PHOSP–COVID study, a prospective longitudinal cohort study of adults discharged from National Health Service hospitals across the United Kingdom after admission for confirmed or clinically diagnosed COVID-19.^19^^,^^20^ Data are accessible to legitimate researchers using the Data and Sample Access Request Form; access to all data and samples collected by PHOSP-COVID are approved by the Core Management Group and Executive board. PHOSP-COVID recruitment was stratified into two tiers, Tier 1 included individuals consenting to information recorded during acute admission and Tier 2 included consent for two follow-up visits post-discharge. Observations were restricted to Tier 2 individuals and initial follow-up data at median 5 months post-discharge; participants were discharged by the end of March 2021 with follow-up available within 240 days of discharge. Individuals withdrawing consent from PHOSP–COVID-19 were excluded. Individuals being managed for an *a priori* diagnosed ILD or pulmonary fibrosis were excluded. Follow-up thoracic CT scans were acquired if the patient remained symptomatic at their clinic appointment and there was concern from the examining physician. Given restrictions in the ability to perform lung function tests during the pandemic (as aerosol generating procedures) and the insensitivity of CXRs in detecting subtle lung damage, there was an impetus to request CT scans based on purely clinical concern. CT scans were not included in the protocol, varied across UK hospital sites, and were identified through the PHOSP–COVID study via linkage to a radiological database. The PHOSP-COVID-19 study was used to collect demographic information from hospital records, including age and sex recorded at birth, as well as clinical characteristics including hospital admission severity based on the level of oxygen support needed, ranging from oxygen supplementation, to continuous positive airway pressure (CPAP) and invasive mechanical ventilation (IMV); chest X-ray records categorised as normal, abnormal (“suggestive of lung fibrosis”, “extensive, persistent changes greater than one-third of lung involvement” and “indeterminate”), or other; lung function measures where available.

### Exposures and outcomes

The primary exposure for those without CT was classification within the RLA at-risk population, whilst for those with follow-up CT, the primary exposure was visually scored RLA equal to or greater than 10% lung involvement. RLA at-risk was defined by percent predicted DL_CO_ <80% and/or abnormal chest X-ray, as previously described.^7^ The first clinically indicated CT performed between 90 days and 240 days post discharge were scored. The presence of RLA on volumetric CTs was scored on a lobar basis by a radiologist blinded to clinical data. The percentage parenchymal involvement of ground-glass opacities (GGO) and reticulation were quantified visually. The sum of the two patterns was averaged across lobes to quantify RLA. Airway dilatation or traction bronchiectasis was not quantified alongside GGO and reticulation as they can be transitory in the acute setting. The outcome was the level of epithelial lung injury biomarkers MMP-7, KL-6, SP-D and SP-A. In secondary analyses, the exposure was specified as the biomarker level whilst RLA classification, percentage lung involvement of GGO, or percentage lung involvement of reticulation, were included as outcomes.

### Epithelial injury biomarker assay

All PHOSP-COVID Tier 2 participants were invited to provide a blood sample at the first follow-up visit, plasma processing and sample prioritisation for biomarker assays were performed as previously described.^5^ Briefly, blood samples were centrifuged and supernatant was aliquoted before being immediately frozen. Samples were provided to Sysmex for the measurement of KL-6, MMP-7, SP-A, and SP-D levels. The biomarker assays were run on a HISCL-800 analyser (Sysmex, Japan), which employs a two-step sandwich immunoassay system to detect antigens in plasma. Biotinylated monoclonal antibodies capture the analyte in the sample and bind to streptavidin-coated magnetic beads, before alkaline phosphatase-conjugated detection with a chemiluminescent readout. All samples were tested after being prediluted eight times and were within the assay's measurement range: 10–6000 U/mL for KL-6, 0.09 ng/mL to 100 ng/mL for MMP-7, 1–1000 ng/mL for SP-A, and 0.02–100 ng/mL for SP-D. Samples passing quality assurance and within limit of quantification were included in analysis.

### Biomarker expression in lung epithelial cells

Gene expression levels of *MMP7* (MMP-7), *MUC1* (KL-6), *SFTPA1* (SP-A) and *SFTPD* (SP-D) were verified in lung epithelial cells from COVID-19 samples (n = 9) compared with non-COVID-19 controls (n = 3) using publicly available spatial transcriptomic data deposited in Gene Expression Omnibus under record GSE190732.^21^ Post-mortem samples from COVID-19 related pneumonia donors were stratified into acute as 1–15 days of duration (n = 3), chronic as more than 15 days of duration (n = 3), and prolonged as 7–15 weeks of duration (n = 3). The Cell Ranger Software Suite (Version 3.1.0) was used to process raw sequencing data with the GRCh38 reference. Spatial RNA sequencing data analysis was performed in R (version 4.5.1) with Seurat (version 5.3.1), cluster annotation was performed using the Human Lung Cell Atlas reference. MMP-7 protein expression in epithelial cells in post-mortem lung tissue from fatal cases of COVID-19 was verified by immunohistochemistry. Briefly, 5 μm thick sections of lung tissue from fatal COVID-19 cases (n = 12) and non-infected control samples (n = 5) collected pre-pandemic from normal adjacent regions of lung during resection surgery were dewaxed and rehydrated. Antigen retrieval was achieved by boiling the sections for 30 min in 10 mM Tris/0.5 M EDTA then stained with anti-MMP7 antibody (Abcam AB205525, 150 μg/mL) overnight. Positive staining was visualised with 3′,3′-diaminobenzidine and sections scanned with a Hamamatsu™ NanoZoomer® S20 digital slide scanner.

### Statistical analysis

To support multivariable modelling and comparable interpretation of non-normal distributions across different concentration units, biomarker levels were log transformed and z-standardised using all samples. The primary analysis tested the difference in mean biomarker levels according to RLA risk classification using unpaired t-tests, an internal replication analysis was performed according to evidence of RLA involvement. Mean values are reported with standard deviations (SD). Non-transformed, absolute biomarker concentrations were also compared using Wilcoxon rank sum tests. Logistic models were specified to calculate and compare the area under the receiver-operator characteristic (AUROC) of biomarkers and admission characteristics for RLA classification and RLA involvement, comparison of AUROC on the same outcome was performed using DeLong's test. Secondary analyses were specified using linear models to test the association of RLA ≥10% on CT relative to <10%, and RLA at-risk relative to low-risk, upon biomarker z-score in unadjusted and adjusted models. Covariates included in adjusted models were age and sex, severe admission (CPAP or IMV), and time difference between sampling and CT; 95% confidence intervals (95% CI) were derived using robust variance estimators. Normality of residuals were used to confirm linearity assumptions. Fractional regression was used to model the percentage involvement of reticulation or GGO according to biomarker z-score, specified as generalised linear models with binomial distribution and logit link. Coefficients are interpreted as the average difference in involvement per increment in z-score. A sub analysis in those with a second clinical CT scored was performed with Wilcoxon Rank Sum for difference in biomarker according to reticulation >5% involvement. Analyses were performed within the Scottish National Safe Haven Trusted Research Environment using Stata 17 MP. Wilcoxon rank-sum was used to test differences in gene expression with cells as the independent unit of analysis, using R.

### Ethics

The PHOSP-COVID study received ethical approval from the Leeds West Research Ethics Committee (20/YH/0225) and is registered on the ISRCTN Registry (ISRCTN10980107), all included participants provided written informed consent. Lung tissue was obtained from postmortem examinations of fatal COVID-19 cases performed at two sites (South Wales and London). Postmortems conducted in South Wales, UK were performed between April and August 2020 (REC 19/NEC/0336). Postmortem tissue collected from London, UK were obtained from Imperial College London Tissue Bank (Project number R20034) from fatal COVID-19 cases. COVID-19 was confirmed by positive SARS-CoV-2 swab either in-life during the patient's final illness or during postmortem in cases of suspected SARS-CoV-2 related death. All non-COVID-19 control tissue was collected from postmortem donors who had died of non-respiratory related causes. All postmortem material was obtained from either consent postmortems or coronial postmortems in which consent for retention of tissue and use in research was obtained from the family or a person in a qualifying relationship.

### Role of funders

PHOSP-COVID is jointly funded by a grant from the Medical Research Council (MRC)-UK Research and Innovation (UKRI) and the Department of Health and Social Care through the National Institute for Health Research (NIHR) rapid response panel to tackle COVID-19 (grant references: MR/V027859/1 and COV0319). The UKILD Consortium was funded by UKRI Ideas to Address COVID-19 (grant reference MR/W006111/1). The views expressed in the publication are those of the authors and not necessarily those of the National Health Service (NHS), the NIHR or the Department of Health and Social Care. The funders had no role in study design, data collection, data analyses, interpretation, or writing of this report.

## Results

**Fig. 1:**
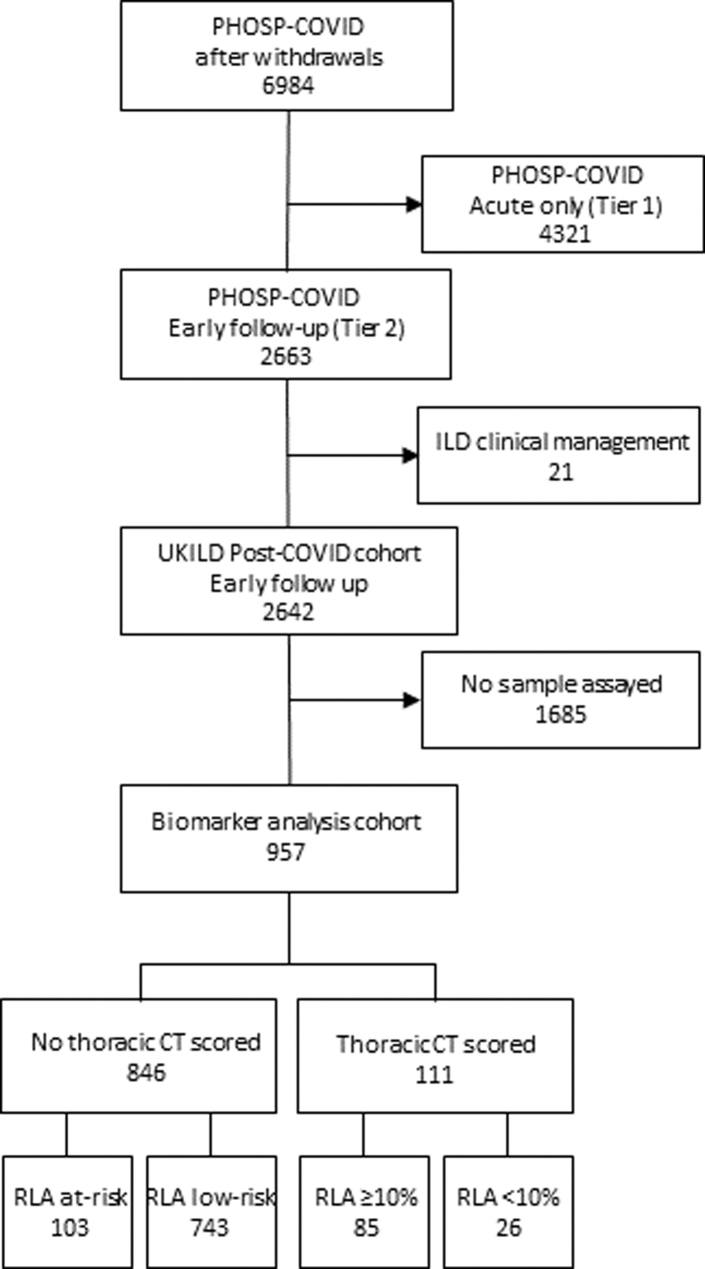
**Flow diagram of participant involvement in UK-ILD Post-COVID epithelial injury study.** Inclusion of participants in the UK-ILD Post-COVID epithelial injury study from the PHOSP-COVID dataset based on early follow-up, no evidence of ILD clinical management, and epithelial injury biomarker assayed.

A total of 957 participants in the UKILD Post-COVID cohort had samples assayed for MMP-7 (n = 955), KL-6 (n = 787), SP-D (n = 957) and SP-A (n = 875), demographics were similar in those who did not have samples assayed (Supplementary Table S1). Of those included in biomarker sampling, 111 had a CT scored (11.6%) and 846 did not have a thoracic CT available (88.4%) (Fig. 1). Between those with CT scored and no thoracic CT available, representation of sex was comparable (n = 70/111; 63.1% and n = 543/846; 64.2%, respectively), age was similarly distributed (58.4 SD 11.2 and 57.0 SD 12.6, respectively) and participants were majority white (n = 73/111; 65.8% and n = 645/846; 76.2%, respectively) (Supplementary Table S2). Acute COVID-19 admission severity was similar between groups (p = 0.059), although there was greater representation of IMV in people with a thoracic CT (n = 35/111; 31.5%) than those without (n = 185/846; 21.9%).

### Biomarker levels and RLA

**Fig. 2:**
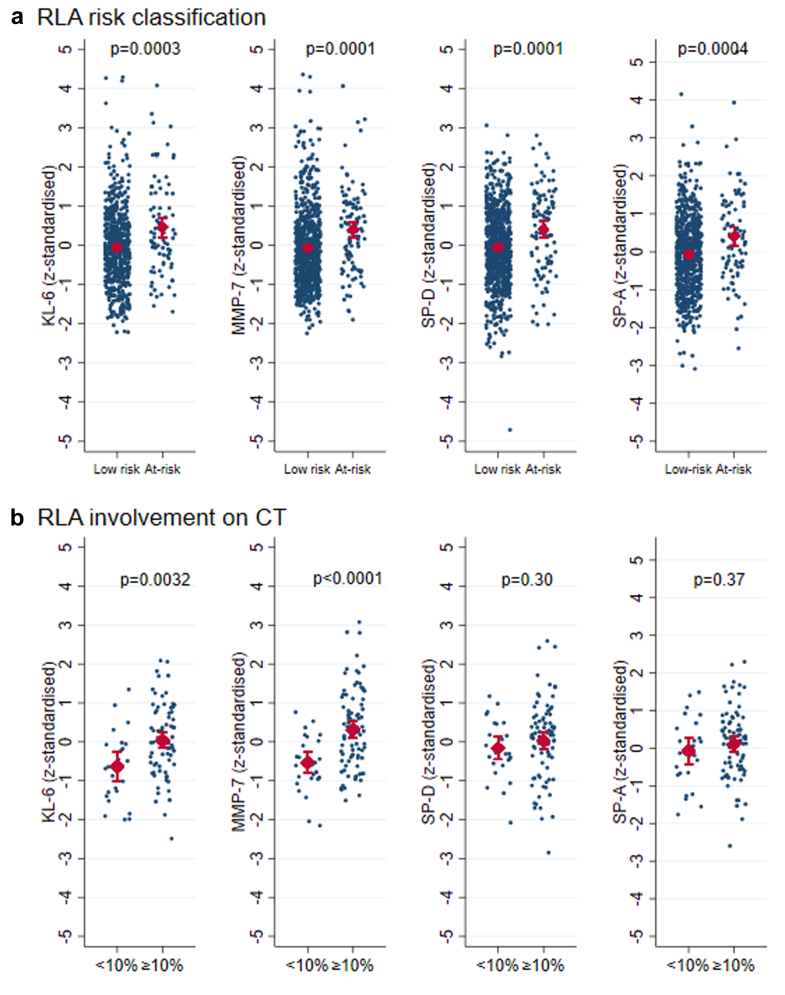
**Comparison of epithelial injury biomarker levels according to residual lung abnormality risk classification and thoracic CT involvement (N = 957).** a) Analysis of z-standardised KL-6, MMP-7, SP-D, SP-A levels between at-risk classification and low-risk classification in those without a thoracic CT (n = 846). b) Replication analysis of z-standardised KL-6, MMP-7, SP-D, SP-A levels between ≥10% residual lung abnormality involvement on thoracic CT and <10% (n = 111). p-values estimated using unpaired t-test, plots present mean values and standard deviation.

A total of 103 people (12.2%) were classified as at-risk of RLA, whilst 742 participants were classified as low-risk (87.8%) (Supplementary Table S3a). Compared with people who were at low-risk, participants classified as at-risk of RLA had greater z-standardised levels of MMP-7 (0.39 SD 1.08 vs −0.07 SD 0.97, p = 0.0001), KL-6 (0.45 SD 1.19 vs −0.04 SD 0.97, p = 0.0003), SP-D (0.40 SD 1.08 vs −0.05 SD 0.99, p = 0.0001), and SP-A (0.41 SD 1.19 vs −0.07 SD 0.97, p = 0.0004) (Fig. 2a). Results were comparable for biomarker concentrations (Supplementary Table S4a).

In replication analysis performed in participants with a CT score, evidence of RLA ≥10% was reported in 85 (76.6%), whilst RLA <10% was observed in 26 (23.4%) (Supplementary Table S3b). Overall, the median time between thoracic CT and sample collection was 16 days (IQR −49 to 76), CT was a median 10 days prior to sampling in those with RLA ≥10% and a median 21 days post sampling in those with RLA <10% (p = 0.090). The mean z-standardised MMP-7 level was greater in those with RLA ≥10% (0.32 SD 1.02 vs −0.54 SD 0.70, p < 0.0001), as well as KL-6 level (0.03 SD 0.86 vs −0.64 SD 0.92, p = 0.0032) (Fig. 2 b). There was no statistical difference in SP-D or SP-A levels between those with RLA ≥10% and those with RLA <10%. Results were comparable for biomarker concentrations (Supplementary Table S4b).

**Fig. 3:**
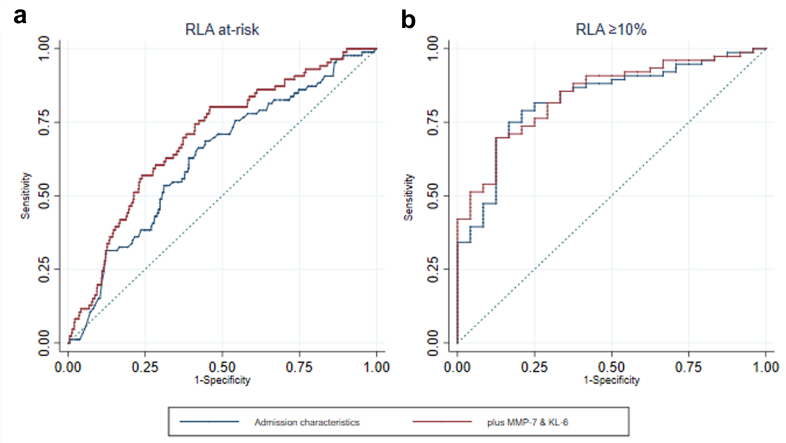
**Residual lung abnormality discrimination with epithelial injury biomarkers (N = 957).** Area Under Receiver Operator Characteristic (AUROC) presented for ability to discriminate a) RLA at-risk participants from low-risk (n = 846) (blue AUROC 0.63 95% CI 0.57–0.69; red AUROC 0.70 95% CI 0.64–0.76; difference p = 0.008), b) RLA ≥10% involvement on thoracic CT from RLA <10% (n = 111) (blue AUROC 0.80 95% CI 0.69–0.91; red AUROC 0.84 95% CI 0.76–0.93; difference p = 0.15). Blue line represents logistic model specified with admission characteristics of age, sex and ventilation status. Red line represents logistic model specified with admission characteristics, plus MMP-7 levels and KL-6 levels.

Admission characteristics of age, sex and admission severity provided an AUROC for RLA at-risk classification of 0.63 (95% CI 0.57–0.69), addition of MMP-7 and KL-6 levels led to an AUROC of 0.70 (95% CI 0.64–0.76), with a significant improvement in performance (p = 0.008). Similarly, addition of MMP-7 and KL-6 improved the performance of admission characteristics in classifying evidence of RLA ≥10% involvement from 0.80 (95% CI 0.69–0.91) to 0.84 (95% CI 0.76–0.93), although this did not reach significance (p = 0.15) (Fig. 3).

### Association between RLA and biomarker level

**Fig. 4:**
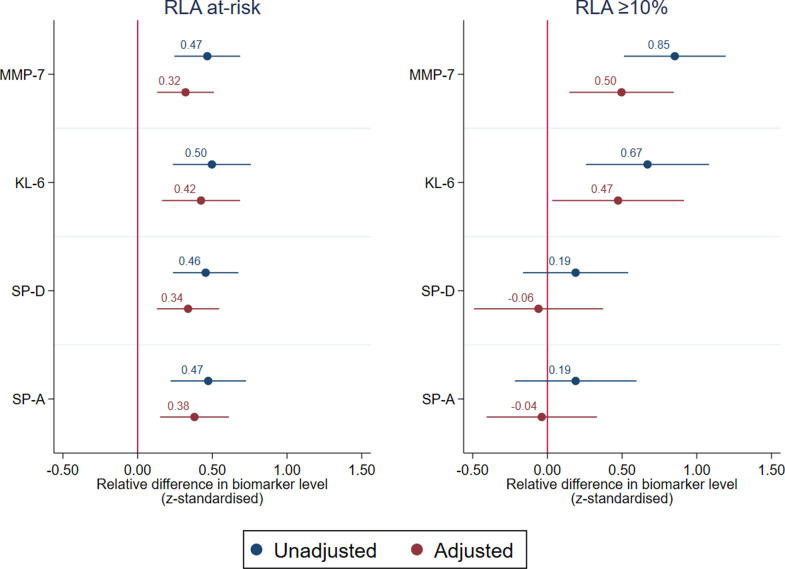
**Difference in biomarker levels according to residual lung abnormality (N = 957).** Unadjusted and adjusted associations modelled with generalised linear model for the difference in z-standardised MMP-7, KL-6, SP-D, SP-A levels for the RLA at-risk classification relative to low-risk classification (n = 846), and RLA ≥ 10% on thoracic CT relative to RLA <10% (n = 111). Covariates in adjusted models include age, sex and severe admission requiring ventilation, presented with 95% confidence interval.

Participants with ≥10% RLA involvement on CT had a 0.85 (95% CI 0.51–1.19, p < 0.001) higher MMP-7 z-score and a 0.67 (95% CI 0.26–1.08, p = 0.001) higher KL-6 z-score, compared with participants with RLA <10% involvement on CT (Fig. 4). Associations were also observed in participants classified as at-risk of RLA relative to low-risk, the MMP-7 z-score was 0.47 (95% CI 0.25–0.69, p < 0.001) higher and the KL-6 z-score was 0.50 (95% CI 0.24–0.76, p < 0.001) higher. No statistically significant association was observed between RLA ≥10% on CT and SP-D (p = 0.30) or SP-A (p = 0.37); whilst RLA at-risk was associated with a 0.46 (95% CI 0.24–0.67) and 0.47 (95% CI 0.22–0.73) higher z-score, respectively, when compared with low-risk participants. Effects were similar when adjusted for age, sex and admission severity.

### Association between biomarker level and RLA involvement

**Fig. 5:**
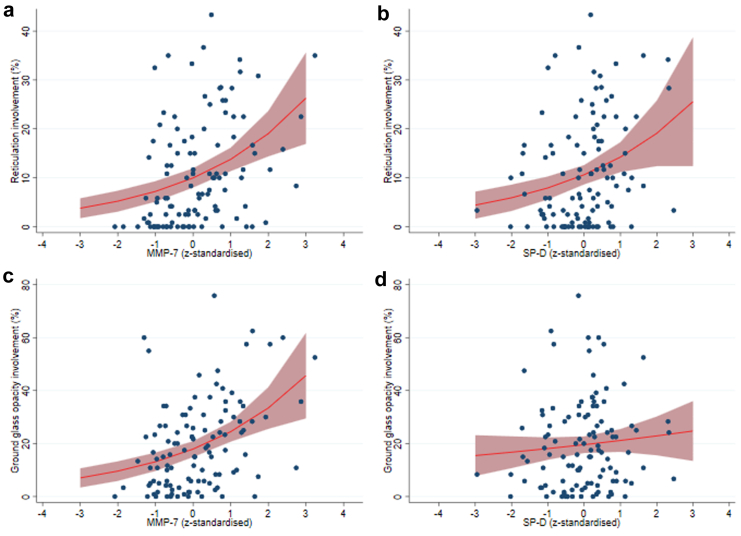
**Association between biomarker level and radiological pattern (N = 111).** Unadjusted association of z-standardised biomarker level and percentage lung involvement, (a) MMP-7 and reticulation, (b) MMP-7 and ground glass opacity, (c) SP-D and reticulation, (d) SP-D and ground glass opacity. Estimates and marginal effects modelled with fractional regression, presented with 95% confidence interval.

In participants with CT scores, a unit increase in MMP-7 z-score was associated with a 3.58% (95% CI 2.04–5.13; p < 0.001) greater involvement of reticulation on average and a 6.25% (95% CI 3.46–9.03; p < 0.001) greater involvement of GGO on average (Fig. 5a, b). A greater KL-6 z-score was similarly associated with percent reticulation (4.80%, 95% CI 2.65–6.96; p < 0.001) and GGO (5.81%, 95% CI 2.66–8.96; p < 0.001). Unit increments in SP-D z-score were associated with a 3.22% (95% CI 1.19–5.24; p = 0.002) greater involvement of reticulation, whilst no statistically significant association was observed with GGO (1.53%, 95% CI −1.39 to 4.46; p = 0.30) (Fig. 5c, d). Similarly, SP-A z-score was associated with more reticulation (3.03%, 95% CI 0.76–5.30; p = 0.009) but not GGO involvement (1.30%, 95% CI −2.01 to 4.61; p = 0.44). Effects were independent of age, sex, admission severity and time between CT and biomarker sampling (Supplementary Table S5).

In a sub analysis of 15 participants who underwent a second CT, median 163 days (IQR) from the initial CT, 301 days (IQR) from discharge and 149 days (IQR) from sampling date, SP-D levels were significantly higher in those with persistent reticulation involvement >5% (Supplementary Table S6).

### Biomarker expression in epithelial cells from COVID-19 lung tissue

**Fig. 6:**
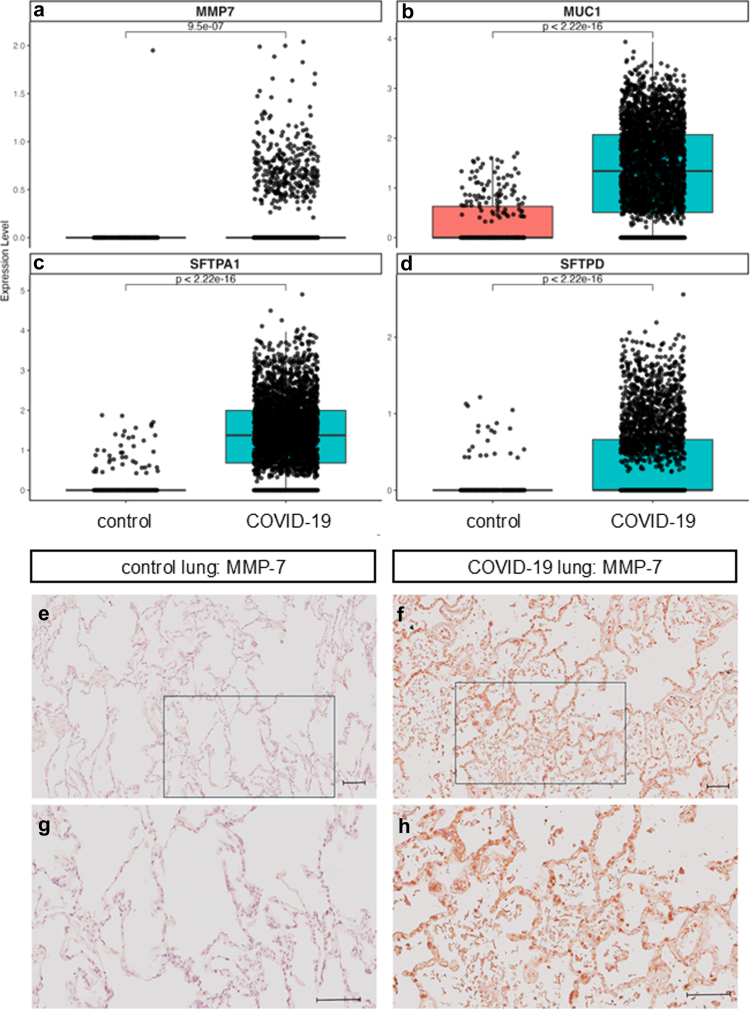
**Epithelial expression of epithelial injury biomarkers in COVID-19 lung tissue.** Publicly available spatial RNA sequencing restricted to cells annotated as lung epithelium represents gene expression of a) *MMP7*, b) *MUC1*, c) *SFTPA1*, d) *SFTPD* in non-COVID-19 control (n = 3) and COVID-19 (n = 9) lung tissue (GSE190732). Box plots present median and interquartile range across conditions, difference tested by Wilcoxon rank-sum. Epithelial MMP-7 protein expression observed in representative images of control (e, g) and COVID-19 injured (f, h) lung tissue. Scale bars at 100 μm, black box in panels e) and f) denote high power regions shown in g) and h), respectively.

Spatial RNA sequencing analysis demonstrated substantially greater gene expression of *MMP7*, *MUC1*, *SFTPA1* and *SFTPD* in epithelial cells from COVID-19 lung tissue than non-COVID-19 controls (Fig. 6a and d). In stratified analysis, greater gene expression levels were robustly observed in chronic and prolonged COVID-19 pneumonia compared with control tissue (Supplementary Figure S1). MMP-7 protein expression was not observed in epithelial cells from non-COVID-19 archive lung tissue (Fig. 6e and g), however levels were clearly observed in alveolar epithelial cells within lung tissue from post-mortem COVID-19 cases (Fig. 6f & h).

## Discussion

People at-risk of RLA according to abnormal chest X-ray and/or impaired gas transfer at follow-up, and those with ≥10% RLA on follow-up CT up-to eight months post-hospitalisation, had greater levels of circulating epithelial lung injury biomarkers. MMP-7 and KL-6, previously validated as pulmonary fibrosis biomarkers,^12^^,^^13^ were greater in those classified as at-risk of RLA compared with low-risk, a finding which was replicated in those with RLA ≥10% on CT compared with <10%. Similarly, MMP-7 and KL-6 levels improved the performance of major admission features in classifying RLA groups. SP-D and SP-A, which are produced predominantly in lung tissues,^22^ were positively associated with involvement of reticulation on CT, but not GGO, suggesting residual lung damage post-COVID-19 that may be representative of fibrotic patterning. In a limited subsample, SP-D levels were greater in participants who had >5% reticulation involvement at a repeat CT. Circulating epithelial lung injury biomarkers may be useful in assessing risk of post-COVID-19 RLA, supporting clinical management in survivors of severe infection and those presenting with long term respiratory symptoms.

MMP-7 is a profibrotic metalloproteinase secreted by epithelial cells in numerous organs, whilst in the lungs it is specifically localised to activated alveolar and bronchiolar epithelial cells.^23^ Localisation of MMP-7 to epithelial cells in COVID-19 lung tissue was verified by immunohistochemistry and spatial transcriptomics. MMP-7 degrades extracellular matrix components, activates cytokines and chemokines, and induces epithelial–mesenchymal transition.^24^ Activity increases in response to tissue injury to enable repair and remodelling, whilst dysregulated activity is linked to increased profibrotic TGF-β signalling and irreversible tissue damage. High circulating MMP-7 levels have been associated with a greater risk of interstitial lung abnormality on CT at 10-years post-sampling and have been proposed as a biomarker of subclinical ILD.^25^ An individual participant data meta-analysis of 10 independent IPF cohorts demonstrated baseline MMP-7 levels were associated with a 23% greater risk of overall mortality and a 27% greater risk of 12-month disease progression, per standardised z-score (increment in standard deviation).^26^ A previous study highlighted median concentrations of plasma MMP-7 of 12.1 ng/mL in IPF and 5.1 ng/mL in healthy controls,^10^ with high sensitivity and moderate specificity of 12.1 ng/mL as a predictive cut-off for all-cause mortality and transplant-free survival. Median concentrations of MMP-7 in those with RLA involvement ≥10% and classified as at-risk were greater than this threshold, suggesting clinically relevant concentrations.

MMP-7 levels have been explored following COVID-19 infection in a number of small cohorts, with higher levels observed in COVID-19 patients compared with controls.^27^ Within COVID-19 patients, higher levels have been observed in individuals who required invasive mechanical ventilation compared with those who did not,^28^ MMP-7 was similarly higher at 2-months post-hospitalisation in severe compared to mild patients, although this discrepancy was not observed at 12-months.^29^ Greater expression of MMP-7 has been observed within epithelial cells and macrophages from bronchoalveolar lavage fluid in severe COVID-19 infection.^30^ By contrast, a plasma proteome study reported lower MMP-7 levels in Long COVID when compared with healthy controls, albeit with greater TGF-β levels and mediators of the integrin activation pathway implicated in profibrotic mechanisms.^31^ Upstream activators of the TGF-β pathway have also been implicated in diffuse alveolar damage following COVID-19.^32^ Although discrepancies in MMP-7 levels by severity have previously been reported, we found that associations of MMP-7 with radiological features in this study were independent of admission severity. Together, these findings implicate MMP-7 as a potentially important prognostic factor for poor recovery and long-term structural changes.

Higher levels of KL-6, SP-A and SP-D have been similarly associated with epithelial cell dysfunction and have all been demonstrated to be upregulated in IPF,^13^^,^^15^ conflicting findings have been reported regarding their association with IPF clinical outcomes of mortality, disease progression and change in forced vital capacity.^26^ Spatial resolution of gene expression demonstrated that *MUC1* (KL-6), *SFPTA1* (SP-A) and *SFPTD* (SP-D) were expressed at greater levels in lung epithelial cells from COVID-19 donors compared with non-COVID-19 controls, which was consistent in chronic and prolonged disease strata.

KL-6 is a mucin-like glycoprotein expressed in regenerating type-II alveolar epithelial cells, released in response to epithelial injury to promote the migration and proliferation of fibroblasts.^33^ Studies of the acute phase of COVID-19 infection demonstrated serum KL-6 as a biomarker of COVID-19 severity and poor clinical outcomes.^34^^,^^35^ In the present study, KL-6 levels were elevated after the acute infection in individuals with greater levels of RLA involvement on CT and in those who were considered at-risk of RLA. KL-6 and MMP-7 performed independently of admission features in classifying RLA risk, supporting these biomarkers as tools for prioritising radiological evaluation in people with ongoing respiratory symptoms after the acute COVID-19 infection.

Surfactant proteins are synthesised and secreted by type-II alveolar epithelial cells to facilitate transport and function of surfactant lipids, playing an important role in immunity and clearance of harmful exposures. Abnormal surfactant protein regulation can trigger apoptosis of regenerative alveolar cells and initiate fibrosis.^36^ Surfactant proteins are produced predominantly within the lung, with leakage into circulation proposed to be indicative of a breakdown in the integrity of the alveolar air-blood barrier. Recent studies have demonstrated an upregulation of SP-D in the acute phase of COVID-19 infection.^37^^,^^38^ The observed relationship between surfactant proteins and reticulation after the acute infection suggests epithelial lung injury that may be consistent with fibrotic damage. An evaluation of thoracic CT changes in 57 severe participants between four and 15 months post-COVID-19 highlighted a decrease in GGO over time that was inversely correlated with reticulation involvement.^39^ Future studies should determine whether longitudinal SP-D levels, and other epithelial lung injury biomarkers, are able to distinguish evidence of active fibrotic mechanisms from residual fibrotic remnants of infection.

A particular strength of this study is the utilisation of a large population of COVID-19 survivors hospitalised with varied severity of acute infection, applying mutually exclusive RLA definitions to address internal replication of biomarker levels. Findings were consistent after adjustment for demographics, severity of ventilation during admission, and time from sampling to CT. Whilst the PHOSP-COVID cohort is a nationwide study, the study was limited by internal replication alone and no healthy control group, people in comparator groups of low-risk of RLA or RLA<10% had evidence of elevated MMP-7 levels, however elevated levels were consistent with the highest levels of radiological involvement. We would propose the measurement of serum MMP-7 to guide the performance of CT and justify risks associated with radiation exposure. Future studies should seek external validation of epithelial injury in individuals with non-resolving abnormalities on CT, including non-hospitalised individuals presenting to Long COVID clinics. Sequencing and immunohistochemistry of post-mortem lung samples from people with COVID-19 related pneumonia, including prolonged disease up to 15 weeks, supported epithelial cells as a source of epithelial injury biomarkers in COVID-19. This work can support reverse translation to preclinical models and in vitro systems for development of potential interventions and therapies targeting persistent mechanisms of epithelial injury. The study design was defined by an *a priori* hypothesis, exploratory studies may identify other important pathways beyond epithelial dysregulation.

There are additional important limitations to our study. Assays reflect circulating biomarker levels, it is possible that lung epithelial cells may not be the sole source of MMP7 production. With the exception of MMP-7, biomarker concentrations in the PHOSP-COVID cohort were largely comparable to healthy controls reported in previous studies,^10^^,^^15^^,^^40^ highlighting that the extent of epithelial injury is not consistent with clinical presentations of ILD. Whilst associations of biomarker levels with RLA risk and involvement were observed, there was considerable variability in effect with a correspondingly modest AUC. Limited evidence of persistent epithelial injury should not be considered evidence of progressive injury, future follow-up and surveillance is necessary to address the natural history of RLA. Mechanical ventilation has been reported to lead to increased levels of epithelial injury biomarkers and fibrotic injury,^41^^,^^42^ which may explain some associations with RLA involvement, however effects were independent of admission severity. The radiological focus of this study was on parenchymal damage, with ground glass opacities and reticulation as the major patterns scored. Traction bronchiectasis is often a transitory feature post the acute setting and was not included in our visual CT score as airway dilatation with infection/inflammation can be reversible. A further limitation was the small number of people with longitudinal data and the small number of samples with spatial transcriptomics available. Future long-term follow-up studies should comprehensively characterise radiological features in post-COVID settings with designs that facilitate longitudinal biobanking. There were discrepancies between the date of sampling and date of clinically indicated CT, which may limit interpretation of injury resolution, although effects were also independent of time differences.

### Conclusion

RLA on thoracic CT, observed beyond three months following COVID-19 related hospital discharge, were associated with circulating epithelial lung injury biomarker levels. Higher levels in individuals at-risk of RLA suggests a substantial number of individuals hospitalised with COVID-19 may be living with subclinical lung injury and profibrotic signalling cascades. Future studies should address the association between temporal changes in fibrotic biomarker levels and the resolution or persistence of radiological lung involvement.

## Contributors

Conceptualization: I.S., J.J., J.C.P., J.M.W., P.L.M., P.M.G., R.J.A., J.F.B., N.C., E.D., L.F., B.G-G., B.G., I.P.H., I.J., S.J., F.K., P.M., J.M., J.E.P., K.P-H., P.R-O., L.C.S., D.J.F.S., M. Sp., L.G.S., S.C.S., A.A.R.T., S.W., A.M., M.P., A.St., S.Y., C.E.B., J.D.C., V.C.H., A.H., M.M., K.P., B.R., R.A.E., R.C.C., L.P.H., R.C.C, L.V.W., R.G.J. Data curation: S.A., E.M.H., M. Se., O.C.L., H.J.C.M., I.S., J.J., N.L., J.K.Q., S.W., M.R.P., A.St., N.G., F.G., A.B.D. Formal analysis: I.S., J.J., J.K.Q., M.P., A.St., B.L., A.L.T., N.G., A.J. Funding acquisition: C.E.B., L.V.W., R.A.E., J.D.C., L.P.H., A.H., M.M., K.P., B.R., O.L., M.R., O.E., H.J.C.M., A.S, M.S., R.S., V.C.H., L.H-W., N.J.G., A.M., S.Y., R.C.C, R.G.J. Project administration: A. Sh., A. Si., A.M., V.Q., A.L.T., A.J., M.R.P. Writing of original draft: I.S., J.J., J.C.P., R.C.C., L.V.W., and R.G.J. Writing and review & editing: I.S., J.J., J.C.P, B.L, A.J., A.L.T, N.G., M.R.P, R.J.A., J.F.B., N.C., E.D., L.F., P.M.G., B.G-G., B.G., I.P.H., L.P.H., I.J., S.J., M.G.J., F.K., P.M., J.M., P.L.M., J.E.P., K.P–H., M.P., V.Q., P.R-O., L.C.S., D.J.F.S., M. Sp., L.G.S., S.C.S., A.A.R.T., S.W., J.M.W., D.G.W., A.B.D., F.G., W.G., E.M.H., N.L., J.Q., A.M., M.P., A.St., S.Y., C.E.B., J.D.C., O.E., R.A.E., N.G., V.C.H., A.H., L.H-W., O.C.L., M.M., H.J.C.M., K.P., M.R., B.R., R.M.S., M. Se., A. Sh., A. Si., R.C.C., L.V.W., R.G.J.

All authors have read an approved the final version of the manuscript. I.S., J.J., and J.K.Q., have accessed and verified the underlying data.

The PHOSP-COVID Collaborative Group provided the underlying data for analysis.

## Data sharing statement

The protocol, consent form, definition and derivation of clinical characteristics and outcomes, training materials, regulatory documents, information about requests for data access, and other relevant study materials are available from www.phosp.org. Spatial transcriptomic data used in this study is publicly available from the Gene Expression Omnibus under record GSE190732.

## Declaration of interests

ASh; AS; AM; BR; DW; DJFS; ED; EH; FG; HM; IJ; JW; JP; KPH; KP; LF; LS; LPH; LHW; LSp; MP; MS; MSp; MRP; MR; MM; MPo; NG; NGr; OE; RA; RS; SW; VQ; VH; WG declare no competing interests.

AART: grants from Heart Research UK, Janssen-Ciliag Ltd, British Heart Foundation. Honoraria and support for attending meetings from Janssen-Cilag Ltd.

AH: support from MRC/UKRI/NIHR (MR/V027859/1), NIHR Manchester Biomedical Research Centre. Chair of NIHR Translational Research Collaboration.

AEJ: stock options and founder for Alevin Therapeutics.

ALT: research grants from Accession Therapeutics. Consulting fees from Accession Therapeutics. Honoraria from Universitas Hasanuddin, Australian Rare Lung Disease. Leadership as Treasurer of British Association for Lung Research. Scientific Advisory Board for Accessing Therapeutics.

ASi: support from MRC/UKRI/NIHR (MR/V027859/1; COV0319).

AD: support from Wellcome CDA 227856/Z/23/Z.

BGG: support from Wellcome fellowship (221680/Z/20/Z).

BG: grants from Alpha-1 Foundation, NIHR Leicester Biomedical Research Centre Respiratory Theme. Consulting fees from Third party anonymised consultancy discussions, GlaxoSmithKline. Honoraria from Limbic. Chair of Midlands ILD Research Alliance. Receipt of reagent from Galecto Biotech.

BL: grants from Action for Pulmonary Fibrosis Mike Bray Fellowship.

CB: support from MRC/UKRI/NIHR (MR/V027859/1; COV0319). Grants from Areteia, Astrazeneca, Chiesi, Genentech, GlaxoSmithKline, Regeneron Pharmaceuticals, Roche, Sanofi. Consulting fees from Areteia, Astrazeneca, Chiesi, Genentech, GlaxoSmithKline, Regeneron Pharmaceuticals, Roche, Sanofi.

FK: consulting fees from Guidepoint.

GJ: grants from Astra Zeneca, Galecto, GlaxoSmithKline, Nordic Biosciences, RedX, Pliant; honoraria from Chiesi, Roche, AstraZeneca, Gilead, GlaxoSmithKline, Boehringer Ingelheim. Consulting fees from AbbVie, AdAlta, Apollo Therapeutics, Arda Therapeutics, Astra Zeneca, Brainomix, Bristol Myers Squibb, Chiesi, Cohbar, Contineum Therapeutics, Galecto, GlaxoSmithKline, Media Therapeutics, RedX, Syndax, Pliant. Payment for expert testimony from Pinsent Masons LLP. Advisory board membership of Boehringer Ingelheim, Galapagos, Vicore. Leadership roles for NuMedii, Action for Pulmonary Fibrosis; Chair of Editorial Board at BMJ Open Respiratory Research.

IS: grants from the Rayne Foundation. Participation on the scientific advisory board for patientMpower Ltd. Honoraria from patientMpower. Support for attending meetings from ERS/ELF Travel grant.

IH: research grant from UKRI, senior investigator award from NIHR, grants from Wellcome Trust. Consulting fees from GlaxoSmithKline. Vice-chair of trustees at Asthma + Lung UK.

JC: grants from Astrazeneca, Genentech, Boehringer Ingelheim, Gilead, Chiesi, Insmed, Grifols, Trudell. Consulting fees from Astrazeneca, Chiesi, GlaxoSmithKline, Insmed, Grifols, Novartis, Boehringer Ingelheim, Pfizer, Janssen, Antabio and Zambon.

JM: Grants from British Heart Foundation Clinical Training Fellowship. Support for attending meetings from institute.

JQ: Grants from UKRI, NIHR, Health Data Research UK, Boehringer Ingelheim, Astrazeneca, Insmed, Sanofi. Consulting fees from GlaxoSmithKline, Sanofi, Chiesi, Astrazeneca.

JPo: Support from UKRI grant to UCL.

JB: support from NIHR BRC (NIHR 203308). Grants from North West Lung Centre.

JJ: grants from Gilead Sciences, GlaxoSmithKline, Microsoft Research, Wellcome Trust, Rosetrees Trus, Chan Zuckerberg initiative, Cystic Fibrosis Trust. Consulting fees from Boehringer Ingleheim, Roche, GlaxoSmithKline, NHSX. Honoraria from Boehringer Ingleheim, Roche, GlaxoSmithKline, Takeda. Patents (GB2113765.8; GB2211487.0). Advisory board for Boehringer Ingleheim, Roche, GlaxoSmithKline.

LVW: support from UKRI (MR/V027859/1), GSK/Asthma + Lung UK (C17-1), NIHR (COV0319), Sysmex OGT. Grants from GlaxoSmithKline, Genentech and Orion Pharma, Wellcome Trust, MRC. Consultancy fees from Galapagos, Boehringer Ingleheim and GlaxoSmithKline. Honorarium from ERJ, MRC Board.

MJ: grants from Wellcome Trust, Asthma + Lung UK, Medical Research Council, Boehringer Ingelheim, AAIR Charity, The Royal Society, GlaxoSmithKline R&D. Consulting fees from Skyhawk Therapeutics.

NC: Consulting fees from Boehringer Ingelheim. Honoraria from Astrazeneca, Boehringer Ingelheim. Payment for expert testimony from National Institute of Health and Clinical Excellence. Advisory board member for DSMB, Insilico, Fortea. Trustee of Pulmonary Fibrosis Northern Ireland, board member of Rare Disease Partnership NI.

NL: director of Research, Intensive Care Society and chair for the Scottish Intensive Care Society Audit Group, Public Health Scotland.

OL: grants from Medical Research Council.

PG: grants from Boehringer Ingelheim. Honoraria from Boehringer Ingelheim, Roche, Teva, Cipla, Brainomix, Astrazeneca, Daiichi-Sankyo, Avalyn. Support for attending meetings from Boehringer Ingelheim, Roche. Advisory board member for GlaxoSmithKline. Stock options for Brainomix, senior medical director of Brainomix.

PLM: grants from Astrazeneca, GlaxoSmithKline, Asthma + Lung UK, Action for Pulmonary Fibrosis. Consulting fees from Hoffman La Roche, Boehringer Ingelheim, Astrazeneca, Trevi, Qureight, Endevour, Redx. Honoraria from Boehringer Ingelheim, Hoffman La Roche. Advisory board member for United Therapeutics.

PRO: Consulting fees from Boehringer Ingleheim, Hoffman La Roche, Chiesi. Honoraria from Boehringer Ingelheim, Hoffman La Roche, Respiratory Effectiveness Group. Support for attending meetings from Boehringer Ingelheim, Respiratory Effectiveness Group. Other fees from Boehringer Ingelheim, Hoffman La Roche, CSL Behring, FibroGen, Vicore Pharma AB, Gilead Sciences, Chiesi, Galecto, Endeavour BioMedicines. Chair of IPF/ILD working group, Respiratory Effectiveness Group. Member of REMAP-ILD committees.

PM: Support from UCL Hospital Biomedical Research Centre, Medical Research Council and GlaxoSmithKline Clinical Training Fellowship.

RE: grants from UKRI/MRC/NIHR, Wolfson Foundation, Genentec/Roche. Consulting fees from Astrazenica/Evidera. Personal speaker fee from Moderna April 2023. Chair of ERS Group 01.02, chair of ATS Pulmonary Rehabilitation Assembly.

RC: support from MRC/UKRI/NIHR (MR/V027859/1; COV0319). Grants from Chiesi. Consulting fees from Arda Therapeutics.

SRJ: support from the NIHR Nottingham Biomedical Research Centre. Grants from Medical Research Council, LAM Action, Fundacio La Marato De TV3, LifeArc. Personal travel award from Ferrer. Charity role as a Trustee of LAM Action.

SY: support from Sysmex Corporation, Oxford Gene Technology IP Ltd. Travel costs from Oxford Gene Technology IP Ltd. Equipment from Sysmex Corporation. Other, former employee of Oxford Gene Technology IP Ltd, which is a Sysmex Group Company.

SS: support from MRC/UKRI/NIHR (MR/V027859/1; COV0319).

PHOSP-COVID Collaborative Group was jointly funded by MRC-UKRI Research and NIHR rapid response panel to tackle COVID-19 (grant references: MR/V027859/1 and COV0319), the UKILD Consortium was supported by UKRI (grant reference MR/W006111/1). The funders had no role in the study design, interpretation or decision to submit for publication.

## Appendix A Supplementary data

Supplementary Material

Consortia membership
