# Supplementary material for: Residual lung abnormality following COVID-19 hospitalisation is characterised by biomarkers of epithelial injury
Source: eBioMedicine. 2026 Jan 24;124:106134. doi: 10.1016/j.ebiom.2026.106134 (PMC12860639; doi:10.1016/j.ebiom.2026.106134)
Supplement: Supplementary Material [file mmc1.docx]

**Residual lung abnormality following COVID-19 hospitalisation is characterised by biomarkers of epithelial injury: Supplementary Material**

Table of Contents

[Supplementary Table 1: Demographics according to those with epithelial injury biomarkers and those not assayed (N=2642) 2](#_Toc204187139)

[Supplementary Table 2: Demographics of participants sampled for biomarker assays with and without thoracic CT available (N=957). 3](#_Toc204187140)

[Supplementary Table 3: Demographics according to residual lung abnormality risk classification and thoracic CT involvement (N=957) 4](#_Toc204187141)

[Supplementary Table 4: Comparison of epithelial injury biomarker levels concentrations according to residual lung abnormality risk classification and thoracic CT involvement (N=957) 4](#_Toc204187142)

[Supplementary Table 5: Unadjusted and adjusted effect of epithelial injury biomarker z-score on involvement of ground glass opacity and reticulation (N=111) 5](#_Toc204187143)

[Supplementary Table 6: Difference in biomarker levels according to persistent reticulation across participants with two CT scans (N=15). 5](#_Toc204187144)

[Supplementary Figure 1: Epithelial expression of epithelial injury biomarkers in COVID-19 lung tissue (N=12) 6](#_Toc204187145)

### **Supplementary Table 1: Demographics according to those with epithelial injury biomarkers and those not assayed (N=2642)**

| **Characteristic** | **Assayed (n=957)** | | **Not assayed (n=1685)** | | |  |
| --- | --- | --- | --- | --- | --- | --- |
| Sex |  |  | |  |  | |
| Male (%) | 613 | 64.1% | | 1012 | 60.1% | |
| Female (%) | 344 | 35.9% | | 673 | 39.9% | |
| Age |  |  | |  |  | |
| Mean (SD) | 57.2 | 12.5 | | 58.3 | 12.6 | |
| Ethnicity |  |  | |  |  | |
| White (%) | 718 | 75.0% | | 1244 | 73.8% | |
| Black (%) | 56 | 5.9% | | 133 | 7.9% | |
| Asian (%) | 131 | 13.7% | | 170 | 10.1% | |
| Other (%) | 30 | 3.1% | | 124 | 7.4% | |
| Index of Multiple Deprivation |  |  | |  |  | |
| Q1-most (%) | 225 | 23.5% | | 384 | 22.8% | |
| Q2 (%) | 196 | 20.5% | | 414 | 24.6% | |
| Q3 (%) | 166 | 17.3% | | 289 | 17.2% | |
| Q4 (%) | 169 | 17.7% | | 288 | 17.1% | |
| Q5-least (%) | 190 | 19.9% | | 301 | 17.9% | |
| Body Mass Index |  |  | |  |  | |
| <25 (%) | 89 | 9.3% | | 122 | 7.2% | |
| 25-<30 (%) | 255 | 26.6% | | 283 | 16.8% | |
| 30-<40 (%) | 369 | 38.6% | | 419 | 24.9% | |
| >=40 (%) | 88 | 9.2% | | 124 | 7.4% | |
| missing | 156 | 16.3% | | 737 | 43.7% | |
| Severity of admission |  |  | |  |  | |
| No O2 (%) | 199 | 20.8% | | 235 | 13.9% | |
| Non-invasive O2 (%) | 344 | 35.9% | | 733 | 43.5% | |
| CPAP (%) | 194 | 20.3% | | 427 | 25.3% | |
| IMV (%) | 220 | 23.0% | | 250 | 14.8% | |
| Chest X-ray |  |  | |  |  | |
| Normal (%) | 362 | 37.8% | | 440 | 26.1% | |
| Other (%) | 62 | 6.5% | | 132 | 7.1% | |
| Abnormal (%)† | 41 | 4.3% | | 80 | 4.7% | |
| Missing | 492 | 51.4% | | 1033 | 61.3% | |
| Restrictive lung function* |  |  | |  |  | |
| ppFVC <80% (%) | 153 | 16.0% | | 271 | 16.1% | |
| ppFVC ≥80% (%) | 384 | 40.1% | | 631 | 37.4% | |
| Missing | 420 | 43.9% | | 783 | 46.5% | |
| Impaired gas exchange* |  |  | |  |  | |
| ppDL_CO_ <80% (%) | 65 | 6.8% | | 107 | 6.4% | |
| ppDL_CO_ ≥80% (%) | 141 | 14.7% | | 179 | 10.6% | |
| Missing | 751 | 78.5% | | 1399 | 83.0% | |
| Lung function* |  |  | |  |  | |
| Mean ppFVC (SD) | 90.1 | 19.7 | | 88.7 | 17.8 | |
| Mean ppDL_CO_ (SD) | 93.2 | 29.6 | | 90.5 | 32.5 | |

*Recorded at early follow-up visit. † “suggestive of lung fibrosis”, “extensive, persistent changes greater than one-third of lung involvement” or “indeterminate”.

### **Supplementary Table 2: Demographics of participants sampled for biomarker assays with and without thoracic CT available (N=957).**

| **Characteristic** | **CT (n=111)** | | **No-CT (n=846)** | | **P-val** |
| --- | --- | --- | --- | --- | --- |
| Sex |  |  |  |  | 0.82 |
| Male (%) | 70 | 63.1% | 543 | 64.2% |  |
| Female (%) | 41 | 36.9% | 303 | 35.8% |  |
| Age |  |  |  |  | 0.22 |
| Mean (SD) | 58.4 | 11.2 | 57 | 12.6 |  |
| Ethnicity |  |  |  |  | 0.054 |
| White (%) | 73 | 65.8% | 645 | 76.2% |  |
| Black (%) | 8 | 7.2% | 48 | 5.7% |  |
| Asian (%) | 24 | 21.6% | 107 | 12.6% |  |
| Other (%) | 6 | 5.4% | 41 | 4.8% |  |
| Index of Multiple Deprivation |  |  |  |  | 0.18 |
| Q1-most (%) | 20 | 18.0% | 205 | 24.2% |  |
| Q2 (%) | 21 | 18.9% | 175 | 20.7% |  |
| Q3 (%) | 16 | 14.4% | 150 | 17.7% |  |
| Q4 (%) | 27 | 24.3% | 142 | 16.8% |  |
| Q5-least (%) | 26 | 23.4% | 164 | 19.4% |  |
| Body Mass Index |  |  |  |  | 0.40 |
| <25 (%) | 9 | 8.1% | 71 | 8.4% |  |
| 25-<30 (%) | 37 | 33.3% | 218 | 25.8% |  |
| 30-<40 (%) | 43 | 38.7% | 326 | 38.5% |  |
| >=40 (%) | 7 | 6.3% | 81 | 9.6% |  |
| missing | 15 | 13.5% | 141 | 16.7% |  |
| Admission severity |  |  |  |  | 0.059 |
| No O_2_ (%) | 25 | 22.5% | 174 | 20.6% |  |
| Non-invasive O_2_ (%) | 36 | 32.4% | 308 | 36.4% |  |
| CPAP (%) | 15 | 13.5% | 179 | 21.2% |  |
| IMV (%) | 35 | 31.5% | 185 | 21.9% |  |
| Chest X-ray |  |  |  |  | 0.15 |
| Normal (%) | 48 | 43.2% | 314 | 37.1% |  |
| Other (%) | 8 | 7.2% | 54 | 6.4% |  |
| Abnormal (%)† | 10 | 9.0% | 31 | 3.7% |  |
| Missing | 45 | 40.5% | 447 | 52.8% |  |
| Restrictive lung function* |  |  |  |  | 0.30 |
| ppFVC <80% (%) | 20 | 18.0% | 133 | 15.7% |  |
| ppFVC ≥80% (%) | 37 | 33.4% | 347 | 41.0% |  |
| Missing | 54 | 48.6% | 366 | 43.3% |  |
| Impaired gas exchange* |  |  |  |  | 0.13 |
| ppDL_CO_ <80% (%) | 11 | 9.9% | 54 | 6.4% |  |
| ppDL_CO_ ≥80% (%) | 21 | 18.9% | 120 | 14.2% |  |
| Missing | 79 | 71.2% | 672 | 79.4% |  |
| Lung function* |  |  |  |  |  |
| Mean ppFVC (SD) | 85.4 | 16.4 | 90.6 | 20.0 | 0.030 |
| Mean ppDL_CO_ (SD) | 89.9 | 33.6 | 93.8 | 28.9 | 0.54 |

*Recorded at early follow-up visit. † “suggestive of lung fibrosis”, “extensive, persistent changes greater than one-third of lung involvement” or “indeterminate”. CT: computed tomography. Q: quintile. O_2_: Oxygen. CPAP: continuous positive airway pressure. IMV: invasive mechanical ventilation. ppFVC: percent predicted forced vital capacity. ppDL_CO_: percent predicted diffusion capacity of the lungs for carbon monoxide. P-values calculated by chi-square or unpaired t-test, accordingly.

### **Supplementary Table 3: Demographics according to residual lung abnormality risk classification and thoracic CT involvement (N=957)**

| **A** | **At-risk (n=103)** | | **Low-risk (n=743)** | | **No CT (N=846)** | | |
| --- | --- | --- | --- | --- | --- | --- | --- |
| Male n(%) | 76 | 74% | 467 | 63% | 543 | 64% | |
| Female n(%) | 27 | 26% | 276 | 37% | 303 | 36% | |
| Mean age (SD) | 58.4 | 12.3 | 56.8 | 12.6 | 57.0 | 12.6 | |
| CPAP or IMV n(%) | 61 | 59% | 303 | 41% | 364 | 43% | |
| **B** | **RLA ≥10% (n=85)** | | **RLA <10% (n=26)** | | **CT scored (N=111)** | | |
| Male n(%) | 57 | 67% | 13 | 50% | 70 | | 63% |
| Female n(%) | 28 | 33% | 13 | 50% | 41 | | 37% |
| Mean age (SD) | 60.2 | 9.9 | 52.6 | 13.5 | 58.4 | | 11.2 |
| CPAP or IMV n(%) | sn | ≥50% | <5 | <20% | 50 | | 45% |

Demographics of participants with A) no CT, and stratified by at-risk or low-risk, and B) CT scored, stratified by RLA involvement ≥10% or <10%. sn: suppressed number

### **Supplementary Table 4: Comparison of epithelial injury biomarker levels concentrations according to residual lung abnormality risk classification and thoracic CT involvement (N=957)**

| **A** | **Low-risk** |  |  | **At-risk** |  |  |  |
| --- | --- | --- | --- | --- | --- | --- | --- |
|  | **n** | **median** | **IQR** | **n** | **median** | **IQR** | **p-val** |
| KL-6 (IU/mL) | 548 | 364.60 | 260.36; 526.44 | 66 | 407.72 | 295.53; 641.50 | 0.037 |
| MMP-7 (ng/mL) | 675 | 12.55 | 9.54; 17.42 | 80 | 17.76 | 10.93; 25.02 | <0.0001 |
| SP-D (ng/mL) | 676 | 45.36 | 29.76; 73.88 | 81 | 69.36 | 39.42; 99.72 | 0.0003 |
| SP-A (ng/mL) | 619 | 29.51 | 21.22; 39.16 | 73 | 34.71 | 25.35; 45.75 | 0.0022 |
| **B** | **RLA<10%** |  |  | **RLA≥10%** |  |  |  |
|  | **n** | **median** | **IQR** | **n** | **median** | **IQR** | **p-val** |
| KL-6 (IU/mL) | 24 | 269.04 | 170.34; 377.58 | 76 | 380.33 | 281.40; 608.06 | 0.0020 |
| MMP-7 (ng/mL) | 26 | 10.27 | 8.21; 13.20 | 85 | 16.38 | 10.78; 22.56 | 0.0001 |
| SP-D (ng/mL) | 26 | 44.10 | 33.27; 62.89 | 85 | 53.99 | 32.80; 70.98 | 0.28 |
| SP-A (ng/mL) | 25 | 31.27 | 22.16; 39.70 | 77 | 33.90 | 24.99; 41.09 | 0.35 |

Difference in biomarker concentrations tested with Wilcoxon rank-sum for A) residual lung abnormality risk classification and B) thoracic CT involvement.

### **Supplementary Table 5: Unadjusted and adjusted effect of epithelial injury biomarker z-score on involvement of ground glass opacity and reticulation (N=111)**

|  | **Biomarker** | **n** | **Unadjusted** | **95%CI** | | **p-val** | | **Adjusted*** | | **95%CI** | | **p-val** | |  |
| --- | --- | --- | --- | --- | --- | --- | --- | --- | --- | --- | --- | --- | --- | --- |
| **Percentage involvement of ground glass opacity** | | | | | | |  | |  | |  | |  | |
|  | KL-6 | 100 | 5.81 | (2.66; 8.96) | | <0.001 | | 3.74 | | (0.78; 6.72) | | 0.014 | |  |
|  | MMP-7 | 111 | 6.25 | (3.46; 9.03) | | <0.001 | | 4.45 | | (1.79; 7.11) | | 0.001 | |  |
|  | SP-D | 111 | 1.53 | (-1.39; 4.46) | | 0.30 | | -0.11 | | (-3.37; 3.15) | | 0.95 | |  |
|  | SP-A | 102 | 1.30 | (-2.01; 4.61) | | 0.44 | | 0.09 | | (-3.50; 3.69) | | 0.96 | |  |
| **Percentage involvement of reticulation** | | | | |  | |  | |  | |  | |  | |
|  | KL-6 | 100 | 4.80 | (2.65; 6.96) | | <0.001 | | 3.73 | | (1.32; 6.14) | | 0.002 | |  |
|  | MMP-7 | 111 | 3.58 | (2.04; 5.13) | | <0.001 | | 2.56 | | (0.97; 4.15) | | 0.002 | |  |
|  | SP-D | 111 | 3.22 | (1.19; 5.24) | | 0.002 | | 2.26 | | (0.01; 4.51) | | 0.049 | |  |
|  | SP-A | 102 | 3.03 | (0.76; 5.30) | | 0.009 | | 2.91 | | (0.29; 5.52) | | 0.029 | |  |

*Covariates include age and sex, severe admission (CPAP or IMV), and time difference between sampling and CT; 95% confidence intervals (95%CI) were derived using robust variance estimators. Estimates and marginal effects modelled with fractional regression.

### **Supplementary Table 6: Difference in biomarker levels according to persistent reticulation across participants with two CT scans (N=15).**

| **Biomarker (z standardised)** | **Reticulation low (n=5)** | | **Reticulation persists (n=10)** | | **P-val** |
| --- | --- | --- | --- | --- | --- |
| KL-6 median (IQR) | -0.92 | -1.11 to -0.03 | -0.35 | -0.71 to 0.46 | 0.28 |
| MMP-7 median (IQR) | 0.34 | 0.02 to 0.59 | 0.11 | -0.60 to 0.74 | 0.68 |
| SP-D median (IQR) | -0.43 | -0.84 to -0.33 | 0.38 | 0.24 to 0.55 | 0.001 |
| SP-A median (IQR) | 0.36 | -0.27 to 0.64 | -0.16 | -0.91 to 1.26 | 0.62 |

Persistent reticulation defined as ≥5% at both CT scans. Low reticulation defined as <5% at second CT scan or >20% relative decline from first CT scan. Median time difference between CT 163 days (IQR 119 to 217), median time of second CT scan from discharge 301 days (IQR 227 to 413), median time of second CT scan from plasma sampling 149 days (IQR 71 to 233).

### **Supplementary Figure 1: Epithelial expression of epithelial injury biomarkers in COVID-19 lung tissue (N=12)**


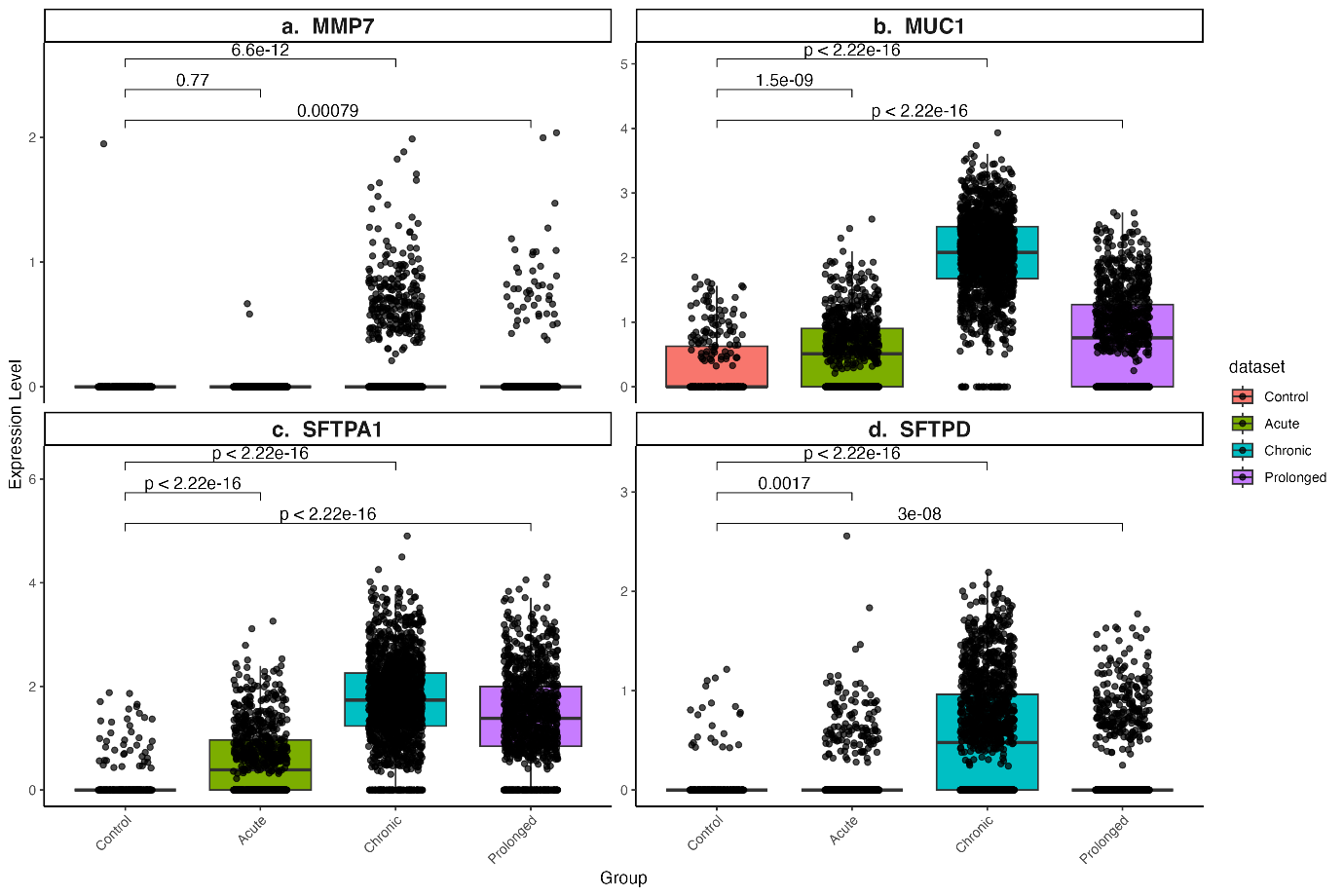


Legend. Publicly available spatial RNA sequencing restricted to cells annotated as lung epithelium represents gene expression of a) *MMP7*, b) *MUC1*, c) *SFTPA1*, d) *SFTPD* in lung tissue from non-COVID-19 control (n=3), COVID-19 acute (1-15 day duration, n=3), COVID-19 chronic (>15 day duration, n=3) and COVID-19 prolonged (7-15 week duration, n=3) participants (GSE190732). Difference relative to control was tested by Wilcoxon rank-sum.
